# The Immorality of “Christian Science”

**Published:** 1899-12

**Authors:** 


### The Immorality of "Christian Science."

"Fools rush in where angels fear to tread."

Knowing nothing of disease, and virtually denying its existence, it should scarcely occasion surprise that the self-styled "Christian Scientist" should boastfully proclaim his ability to cure the sick and heal the maimed. So deluded have some of the disciples of the cult become, that they are permitting their zeal to carry them beyond the bounds of possibility. With their beliefs we have nothing to do, but when they promulgate the hope, as did a public speaker recently in Philadelphia, that "cancer, consumption, Bright's disease, blindness, typhoid fever, curvature of the spine, locomotor ataxia, valvular disease of the heart and the alcohol and drug habits" can be cured by "Christian Science," tolerance is exhausted, and steps should be taken to restrain the rabid utterances and the irrational practices of such ignorant and irresponsible persons. Liberty is one thing, and license another, and the crime of even suggesting such obviously false doctrines and immoral practices should be prevented by severe punishment. Those who speak thus should know better, or their ignorance is criminal. Human life must still be considered as too serious and too valuable to be thus lightly dealt with.—*The Journal.*

